# The Emerging Role of PIWI-Interacting RNAs (piRNAs) in Gastrointestinal Cancers: An Updated Perspective

**DOI:** 10.3390/cancers14010202

**Published:** 2021-12-31

**Authors:** Ismael Riquelme, Pablo Pérez-Moreno, Pablo Letelier, Priscilla Brebi, Juan Carlos Roa

**Affiliations:** 1 Institute of Biomedical Sciences, Faculty of Health Sciences, Universidad Autónoma de Chile, Temuco 4810101, Chile; ismael.riquelme@uautonoma.cl; 2 Millennium Institute on Immunology and Immunotherapy, Department of Pathology, School of Medicine, Pontificia Universidad Católica de Chile, Santiago 8380000, Chile; pablo.perezm@uc.cl; 3 Precision Health Research Laboratory, Departamento de Procesos Diagnósticos y Evaluación, Facultad de Ciencias de la Salud, Universidad Católica de Temuco, Manuel Montt 56, Temuco 4813302, Chile; pletelier@uct.cl; 4 Millennium Institute on Immunology and Immunotherapy, Laboratory of Integrative Biology (LIBi), Center for Excellence in Translational Medicine—Scientific and Technological Bioresource Nucleus (CEMT-BIOREN), Universidad de La Frontera, Temuco 4810296, Chile; priscilla.brebi@ufrontera.cl

**Keywords:** PIWI-interacting RNAs (piRNAs), gastrointestinal cancers (GI cancers), prognostic biomarkers, diagnostic biomarkers, therapeutic targets

## Abstract

**Simple Summary:**

Gastrointestinal (GI) cancers are high mortality malignancies due to late diagnosis, the presence of metastasis and drug resistance development. Novel and more reliable biomarkers and therapeutic targets are still needed for these diseases. PIWI-interacting RNAs (piRNAs) are small transcripts that are involve in gastrointestinal carcinogenesis and have been proposed as promising diagnostic or prognostic biomarkers and as potential therapeutic targets in these malignancies. This review describes important topics about piRNAs including their molecular characteristics, biosynthesis processes, gene expression silencing mechanisms, and the manner in which these transcripts have been studied in samples and cell lines of GI cancers. In addition, this article discusses the potential clinical usefulness of piRNAs as biomarkers and therapeutic targets in GI cancers.

**Abstract:**

Gastrointestinal (GI) cancers produce ~3.4 million related deaths worldwide, comprising 35% of all cancer-related deaths. The high mortality among GI cancers is due to late diagnosis, the presence of metastasis and drug resistance development. Additionally, current clinical markers do not adequately guide patient management, thereby new and more reliable biomarkers and therapeutic targets are still needed for these diseases. RNA-seq technology has allowed the discovery of new types of RNA transcripts including PIWI-interacting RNAs (piRNAs), which have particular characteristics that enable these molecules to act via diverse molecular mechanisms for regulating gene expression. Cumulative evidence has described the potential role of piRNAs in the development of several tumor types as a likely explanation for certain genomic abnormalities and signaling pathways’ deregulations observed in cancer. In addition, these piRNAs might be also proposed as promising diagnostic or prognostic biomarkers or as potential therapeutic targets in malignancies. This review describes important topics about piRNAs including their molecular characteristics, biosynthesis processes, gene expression silencing mechanisms, and the manner in which these transcripts have been studied in samples and cell lines of GI cancers to elucidate their implications in these diseases. Moreover, this article discusses the potential clinical usefulness of piRNAs as biomarkers and therapeutic targets in GI cancers.

## 1. Introduction

Gastrointestinal (GI) cancers comprise 26% of the global cancer incidence and 35% of all cancer-related deaths. In 2018, there were ~4.8 million new cases of GI cancers and ~3.4 million related deaths worldwide [1]. The main reasons for the high mortality of GI cancers are (1) the late diagnosis due to non-specific abdominal symptoms and the lack of validated screening programs in the most affected countries, which results in few individuals that can benefit from surgery, the treatment with the best prognosis; (2) the development of metastasis that induces failure in other distant organs; and (3) the development of intrinsic or acquired drug resistance in primary and metastatic tumor cells that affects therapeutic response and increases the recurrence rates and death in patients [2,3,4,5].

GI cancers are routinely studied through direct or indirect imaging of tumors and by blood biochemical markers that have proven to be relevant in complementing diagnosis and prognosis more than being pivotal for the diagnosis itself. The commonly used blood biochemical markers are total bilirubin, aspartate aminotransferase (ASAT), alanine aminotransferase (ALAT), and tumor markers such as cancer-related antigen 72.4 (CA72.4), carcinoembryonic antigen (CEA), carbohydrate 19-9 (CA19-9), and alpha-fetoprotein (AFP), among others [4,6,7,8]. For instance, serum levels of tumor markers CEA and CA19-9 are usually elevated in GI cancers and serve as a standard for clinical diagnosis, although none has sufficient sensitivity or specificity to be used in a differential diagnosis and early-stage detection [4,6,7,8]. Given these limitations, diagnosis occurs mainly by pathological examination of the biopsy when the disease is advanced.

In addition, as most GI cancers are complex and heterogeneous malignancies both histologically and genetically, patient outcome is difficult to predict using the classical morphologic and molecular criteria. The prognosis and predictive capacity of classical classification systems do not adequately guide patient management, thereby new and more reliable biomarkers and therapeutic targets are still needed for these diseases. Fortunately, recent advances in high-throughput technologies have led to the discovery of new, promising molecules that might help to overcome the diagnostic, prognostic, and therapeutic complications observed in GI cancers.

In recent years, next-generation sequencing (NGS) technologies have helped to discover new types of housekeeping and regulatory RNAs in the cells. Only ~2% of the whole human genome is transcribed and translated into polypeptides or proteins, and the remaining ~98% of the genome is transcribed into non-coding RNAs (ncRNAs), which do not encode for polypeptides or proteins [9,10]. This group of ncRNAs is composed of two main subgroups: (1) those RNA molecules having sequences longer than 200 nucleotides (nts), called “large ncRNAs” that include *long non-coding RNAs* (lncRNAs), *circular RNAs* (circRNAs), etc.; and (2) transcripts of shorter nucleotide sequences, called “small ncRNAs” such as *small interfering RNAs* (siRNAs), *small nucleolar RNAs* (snoRNAs), *microRNAs* (miRNAs), and *PIWI-interacting RNAs* (piRNAs) [11,12]. Interestingly, each of these transcripts has different biosynthesis processes, acts via diverse molecular mechanisms to regulate gene expression, and thus participates in multiple biological functions in the cells [9,13].

Particularly, the *P-Element-induced wimpy testis (PIWI)-interacting RNAs* (piRNAs) were first described in 2001 in experiments using *Drosophila* models evidencing their role in fertility [14]. Then, piRNAs were involved in the function of mammalian germ cells [15]. During the last two decades, researchers have described piRNAs as small RNA molecules able to bind PIWI proteins to form piRNA/PIWI complexes, which act as mediators in several processes including transposon silencing, spermiogenesis, genome rearrangement, epigenetic regulation, protein regulation, and germ stem-cell maintenance in both normal and abnormal cells [16]. In the last years, several reports have evidenced that piRNAs are involved in the carcinogenesis of different malignancies due to their epigenetic effect on the different processes that trigger the altered gene expression observed in tumor cells, which has emerged as one of the likely explanations for all genomic abnormalities and signaling pathways’ deregulations observed in cancers [17,18,19]. In addition, these piRNAs might be also considered as promising diagnostic or prognostic biomarkers or as potential therapeutic targets in malignancies [20,21].

This review describes important topics about piRNAs such as molecular characteristics, biosynthesis processes, gene expression silencing mechanisms, and how these interesting transcripts have been studied in samples and cell lines of GI cancers to elucidate their roles in these diseases. Moreover, this article discusses the potential clinical usefulness of piRNAs as potential biomarkers and therapeutic targets in GI cancers.

## 2. Molecular Characteristics of piRNAs

As mentioned, PIWI-interacting RNAs (piRNAs) constitute a class of small ncRNAs. These small ncRNAs can bind certain proteins, including proteins belonging to the Argonaute family, to form the RNAse-induced silencing complex (RISC) and together recognize the target mRNA through base pairing. Subsequently, the Slicer endonuclease activity of Argonaute proteins cleaves target transcripts to accomplish gene silencing [22].

However, piRNAs have some specific differences compared to other types of small ncRNAs such as miRNAs and siRNAs. (1) The miRNAs and siRNAs are molecules of 20–22 nucleotides (nt); meanwhile, piRNAs are slightly longer molecules (24–31 nt) with 5′-terminal uridine or 10th position adenosine bias and a 2′-O-methyl modification site at the 3′ end that lacks clear secondary structure motifs [23]. (2) Proteins belonging to Argonaute family that compose RISC have two main subfamilies: the subfamily of Argonaute (AGO) proteins that are ubiquitously expressed in the cells and the subfamily of PIWI proteins that are mainly expressed in germline cells [24] but that can be also expressed in somatic cells. The miRNAs and siRNAs bind mainly AGO proteins to form RISC; meanwhile, piRNAs can bind PIWI proteins to form specific RISCs (also known as piRISCs or piRNA/PIWI complex) [22,25]. (3) The precursors of miRNAs and siRNAs are double-stranded RNA molecules that are processed through an RNase type III enzymes (known as Dicer); meanwhile, piRNAs are processed from single-stranded RNA precursors transcribed from intergenic regions termed piRNA clusters via a Dicer-independent mechanism [26,27].

These molecular characteristics of piRNAs and the processes that form them are still being elucidated, so this knowledge has been periodically updated. In addition, the multiple processing mechanisms, the accompanying proteins, and the great variety of transposon sources convert piRNAs (and, hence, piRISCs) into more diverse molecules than any other known class of cellular RNAs, constituting, at the moment, the greatest class of non-coding RNAs [25,27].

## 3. Biogenesis of piRNAs

There are two main processes to generate piRNAs: (1) the processing pathway of primary piRNAs and (2) the ping-pong cycle that amplifies secondary. The primary piRNAs are originated from three types of genomic sequences, the single-stranded piRNA clusters, dual-stranded piRNA clusters, or piRNA genes, and exert their silencing functions primarily within the nucleus. Molecularly, primary piRNAs are characterized by having uridine (U) at their 5′ end [25]. In the case of secondary piRNAs, they are mainly transcribed from dual-stranded piRNA clusters or transposon sequences and exert their regulatory functions mainly in the cytoplasm. The structure of secondary piRNAs are characterized by showing a 10-nt complementarity with primary piRNAs at their 5′ ends and by possessing both a sense bias and 10A bias (the presence of adenosine at the 10th nucleotide) [28].

### 3.1. Biogenesis of Primary piRNAs

As mentioned, the transcription of primary piRNAs occurs mainly from single-stranded piRNA clusters, dual-stranded piRNA clusters, and piRNA genes. The transcription mechanisms exerted by piRNA genes and single-stranded piRNA clusters are similar and involve the transcription of the piRNA precursor (pre-piRNAs) after the corresponding activation of nearby histones through histone H3 lysine 4 dimethylation (H3K4me2). The transcription complex includes promoter elements, RNA polymerase II (RNA pol II), and downstream components [29]. Briefly, in heterochromatin, protein Rhino and its related protein Deadlock recruit transcription factors such as Moonshiner protein and RNA polymerase II to form the assembly of the pre-initiation complex (PIC) that initiates the pre-piRNA transcription [29,30]. Then, this nascent pre-piRNA undergoes 5-terminal capping, 3-terminal polyadenylation, and sometimes selective splicing [29]. In the case of dual-strand clusters, they can transcribe piRNAs from both genomic strands, depending on promoters of nearby coding genes, as well as their transcripts not always being processed in the same manner [28,31].

Once the long sequence of this pre-piRNA is transcribed and submitted to capping, polyadenylation, or other processes [29], this transcript is transported out of the nucleus through nuclear pores and is aggregated in the Dot COM site of the perinuclear structure [32] to bind then with Yb bodies in the cytoplasm near to mitochondria [33]. The protein components of Yb bodies (e.g., Armitage) act to enhance the assembly capacity of pre-piRNAs and PIWI [33]. In addition, in the mitochondria surface, the 5′ end of pre-piRNA is modified by incision via ribonuclease Zucchini (Zuc) along with its co-factor Minotaur (Mino), resulting in a piRNA intermediate containing uracil in its 5′ end [34,35]. PIWI protein contains a group of conserved domains including the PAZ domain that is able to recognize piRNA intermediate through uracil in the 5′ end to form the precursor complex of piRISC [36]. This piRISC precursor is cut by endonuclease Zuc to release one or more piRISCs into the cytoplasm. Those separated piRISCs can take two ways to finish their maturation: (1) ribonuclease Zuc can exert the cleavage of the 3′ end of the piRNA from piRISC or (2) piRISC can be processed via the Papi/Trimmer-dependent manner [34,37,38]. In this last mechanism, Papi protein can recruit the Trimmer enzyme, which binds the Tudor domain at the N-terminus of the PIWI protein to form another cleavage complex to modify the 3′ end of piRNA [37,39]. Finally, Hen1 catalyzes the 2′-O methylation at the 3′ end [25].

When the piRISC maturation process is finished, this complex is transported into the nucleus through nuclear pores to repress the transcription of target genes by sequence complementarity [40].

### 3.2. Biogenesis of Secondary piRNAs

Secondary piRNAs are mainly transcribed from dual-stranded piRNA clusters or transposon sequences to be then processed by other members of the PIWI subfamily, such as Aubergine (Aub) and Argonaute3 (AGO3), through the mechanism known as the ping-pong cycle [41,42]. In this process, Aub and AGO3 act together in the cytoplasm to split sense and antisense transposon transcripts through their Slicer activity, transforming to these sequences, after a series of not-well-defined steps, into secondary piRNAs or more precisely into piRISCs/AGO3 and piRISCs/Aub complexes [42,43]. Therefore, since this mechanism provokes the consumption of transposon transcripts, the result will be the silencing of transposons [25,28].

The Slicer activity directs the cleavage of its cognate target RNAs across the position between 10 and 11 nt, measured from the 5′ end of the associated small RNA [25]. Thus, reciprocal cleavage of transposon transcripts by piRNA/AGO3 and piRNA/Aub complexes determines the 5′ end of secondary piRNAs; however, the formation of their 3′ terminus is still unknown [25]. Those Aub-bound primary piRNAs have an antisense and 1U (1 Uridine) bias and, on the other hand, the AGO3-bound secondary piRNAs present a 10A (10 Adenosine) bias and have 10-nt complementarity at their 5′ ends with the Aub-bound piRNAs. This interaction between piRNAs with 1U and piRNAs with 10A that have a 10-nt 5′ overlap in the pathway is often called the ping-pong signature [25,42,43].

**Figure 1:**
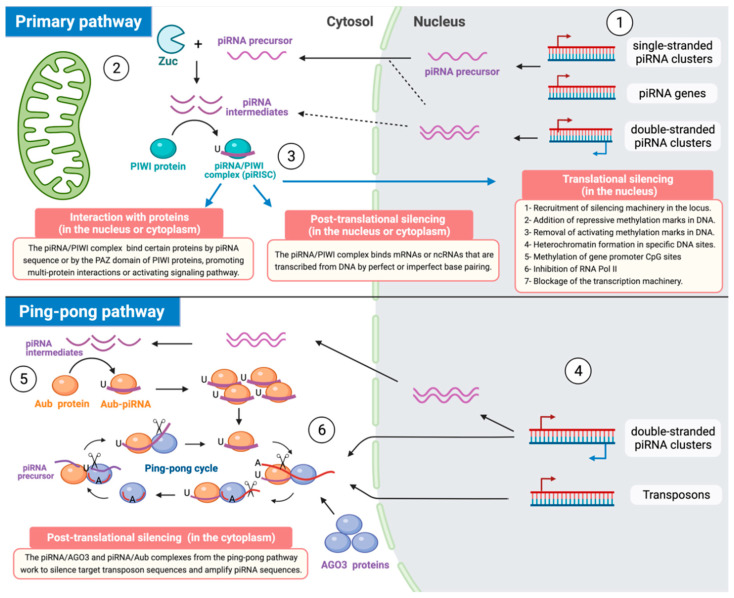
The biogenesis of piRNAs consists of two main processes the primary and the ping-pong pathways. In the primary pathway, (1) piRNAs’ precursors (pre-piRNAs) are transcribed from genomic regions such as piRNA clusters (single- and double-stranded) and piRNA genes by RNA Pol II. Then, (2) pre-piRNAs are processed by Zuc and other proteins to form piRNA intermediates to be then loaded onto PIWI protein. Then, (3) these primary piRNAs (piRISCs) are edited once again to finish the maturation process before starting their gene-silencing activity or protein interacting in both the nucleus and cytoplasm. In the ping-pong pathway, (4) piRNAs proceed from dual-stranded sequences or transposons. Then, (5) the sequences are processed in different manners, binding Aub protein, for example. Finally, (6) piRNA/Aub and piRNA/AGO3 complexes work together in the cytoplasm to silence the target transposon sequences and amplify piRNA sequences at the same time.

Despite several things related to the ping-pong route that have been recently discovered, especially in other species, there are still many other issues to be elucidated concerning this process. However, evidence has been demonstrating that secondary piRNAs (piRISCs/AGO3 or pi-RISCs/Aub) exert their post-transcriptional gene silencing control in the cytoplasm mainly by cleaving transposons [44,45] (Figure 1).

## 4. The Functions of the piRNA/PIWI Complex

PiRNAs can bind PIWI proteins to form piRISCs, which can influence transposon silencing, genome rearrangement, epigenetic regulation of gene expression, and protein regulation. Therefore, these molecules can be involved in physiological processes such as germ stem-cell maintenance, gametogenesis, etc., and in pathological processes such as carcinogenesis.

### 4.1. Functions of the piRNAs/PIWI Complex in the Transcriptional Gene Silencing of Transposons and Other Genes

In mammalian cells, there are many repetitive pieces of DNA named transposons (also known as transposable elements (TEs)), which can move within the genome (a phenomenon called transposition), exerting a series of actions to modify the cell phenotype [46]. These TEs can be subgrouped into two major classes: (1) DNA transposons, which can excise themselves from the genome, move as DNA, and insert themselves into new genomic sites, and (2) Retrotransposons, which are DNA transposons originated from RNA intermediates that were first transcribed and then reverse transcribed to be finally inserted at new genomic locations [47].

In general, the uncontrolled expression and transposition of TEs are usually considered to be a threat to the genome integrity because these elements can be independently copied or moved from their original position to be then inserted into an atypical site of the genome, producing interference with normal sequences and significantly increasing the risk of developing gene mutations [48,49]. Since piRNA clusters harbor a large number and variety of transposons, it is easy to assume that piRNAs are able to regulate primarily the activity of these transposons. For this reason, the piRNA/PIWI complexes (or piRISCs) are usually considered as “the immune system of the cells” as they regulate the expression levels and transposition capability of TEs across the genome [50,51].

Since DNA transposons are currently not mobile in the human genome [47], the transposon silencing effect of piRNA/PIWI complexes is achieved mainly by repressing the expression of retrotransposon RNAs at both transcriptional and posttranscriptional levels [44]. Transcriptional silencing is mediated by nuclear piRISCs that contain PIWI proteins (primary piRNAs), whereas posttranscriptional silencing is mediated by cytoplasmic piRISCs that contain Aub or AGO3 proteins (secondary piRNAs) [51].

In transcriptional silencing, PIWI proteins of piRNA/PIWI complex combine with downstream effectors Panoramix (Panx/CG9754) and Asterix (DmGTSF1) to induce transcriptional gene suppression by recruiting silencing machinery components on the source locus [44]. Other downstream effectors are the protein Eggless (Egg) and its co-factor Windei (Wde), which add the repressive H3K9me3 marks to the target DNA region; meanwhile, lysine-specific demethylase 1 (Lsd1) removes the activating H3K4me2 marks from DNA promoter regions, causing the subsequent inhibition of RNA Pol II transcription [52]. Then, heterochromatin protein 1 (HP1) provokes heterochromatin formation in specific DNA sites that prevents the functioning of the transcription machinery. At the same time, piRNA/PIWI complex recruits DNA methyltransferase (DNMT) to methylate CpG sites of specific genes located in nearby regions that reduce their probability to be transcribed [53,54]. Probably, there are other simultaneous and synergic mechanisms but many of them are still unknown.

In summary, evidence demonstrates that the transcriptional gene silencing induced by piRNA/PIWI complexes involves mechanisms of chromatin modeling, DNA methylation changes, and blockage of the transcriptional machinery. Therefore, the reduced expression of piRNA/PIWI complex members (or their effectors) or the loss of piRNA/PIWI complex function (or their effectors) can induce greater genomic damage via the unrestricted action of TEs, along with triggering an abnormal expression of other disease-related genes. The transcriptional deregulation of transposons and other genes (e.g., oncogenes or tumor-suppressor genes) has been commonly described in pathological situations such as fertility problems and carcinogenesis [19,31,40,55,56].

### 4.2. Functions of piRNAs/PIWI Complex in the Post-Transcriptional Gene Silencing

In the post-transcriptional control of gene expression, piRNA/PIWI complexes can bind to coding RNAs (mRNAs) or non-coding RNAs (e.g., lncRNAs, pseudogenes, etc.) that are transcribed from DNA [54]. Here, the piRNA/PIWI complexes bind target RNA through effective piRNA:RNA interactions formed by a strict base pairing within 2–11 nt at the 5′-end of the piRNA that is part of the piRISC (perfect pairing) or by a less strict base pairing within 12–21 nt (imperfect pairing) [57], in a similar manner as occurs with the miRNA silencing mechanism. When base pairing is done, the proteins that are part of the RISC portion of piRISC, that is, comprised of different types of protein among cell types and species, work together in order to produce cleavage or deadenylation of the target RNA, which will result in target RNA decay [58,59].

This same mechanism of post-transcriptional silencing can be also exerted on TEs to maintain genome integrity [58,60]. In fact, the ping-pong mechanism for piRNA amplification uses a similar process to selectively detect and slice the RNAs from transposons, inducing a post-transcriptional silencing of TEs [28,50].

### 4.3. Functions of piRNAs/PIWI Complex in the Interaction with Proteins

The piRNA/PIWI complexes can also directly bind some proteins either through piRNAs or through the PAZ domain from PIWI proteins. This relationship between piRISCs and proteins can promote multi-protein interactions within cells, leading to the activation of a specific signaling pathway. For example, piRISCs can also influence importantly in the subcellular localization of the protein, producing unexpected loss or gain function [40,54,61]. Interestingly, as will be described next, these piRISC-protein interactions can directly or indirectly trigger the activation of pro-carcinogenic proteins or the inactivation of tumor-suppressor proteins, as occurs in most cancer tissues, via some post-translational modifications such as phosphorylation, among others [16,62].

## 5. The Role of piRNAs in Gastrointestinal (GI) Cancers

As previously mentioned, GI cancers encompass ~26% of cancer incidence and 35% of all cancer-related deaths worldwide [1], becoming global public health challenges due to their high mortality generated because of late diagnosis, metastasis development, and drug resistance [2,3,4,5]. In addition, routine markers such as CA19-9 and CEA are not accurate tools for early diagnosis, prognosis, and follow-up purposes. Therefore, more reliable biomarkers and therapeutic targets are still required for these malignancies. Fortunately, high-throughput technologies, bioinformatic platforms, and novel molecular biology techniques have helped to discover and evaluate the usefulness of new promising molecules to overcome the diagnostic, prognostic, and therapeutic issues observed in GI malignancies. Table 1 reviews different piRNAs that have been studied as biomarkers and/or cancer regulators in the tissues of patients with GI tumors.

### 5.1. Gastric Cancer (GC)

Gastric cancer (GC) is the fifth most frequently diagnosed cancer and the third most lethal cancer worldwide. Each year, about 1 million new cases of GC are diagnosed and more than 700,000 people die because of this disease, thereby representing ~10% of the cancer-related deaths in the world [76]. GC is a multifactorial disease, where certain environmental and genetic factors can increase the risk of developing this neoplasm, e.g., *Helicobacter pylori* infection (the most important risk factor), Epstein-Barr virus (EBV) infection, diet, alcohol consumption, smoking, and family history of GC (mainly linked to *CDH1* gene alterations) [77]. The poor prognosis of GC and the need for early diagnostic markers for this malignancy are evidenced in the fact that most of the patients are diagnosed in an advanced stage where the rate of median survival is less than 12 months [78].

#### 5.1.1. The piRNAs as Prognosis Markers and Regulators of Gastric Cancer (GC)

Several piRNA-related studies have suggested that these transcripts can be implicated in the development and/or prognosis of GC. For example, Chen et al. described a microarray analysis carried out in four samples of GC tissues and four paired non-tumoral adjacent tissues (NAT), in which piR-651 was found to be upregulated in GC tissues. These results were validated in another sample cohort, evidencing those levels of piR-651 were even higher in those tumors with greater TNM stages. Interestingly, Chen et al. also found that piR-651 expression was higher in samples of colon, lung, and breast cancer, as well as in cell lines of hepatic carcinoma (HepG2), cervical cancer (HeLa), breast cancer (Bcap-37), mesothelioma (MSTO211H), lung cancer (NCI-H446), and gastric cancer (MGC-803 and SGC7901). Subsequently, the piR-651 expression was modulated via an inhibitor transfected into two GC cells lines (MGC803 and SGC-7901 cells) to evaluate how this transcript influences the cell growth and cell cycle in these cells. The results confirmed that piR-651 promotes cell growth in GC cells by inhibiting the cell cycle arrest at the G2/M phase. Therefore, piR-651 could be potentially involved in the development of GC and could be considered as a candidate diagnostic marker for GC [63].

Later, Cheng et al. published another study in which piR-823 expression was found repressed in GC tumors and GC cell lines MGC-803 and SGC-7901 compared to the corresponding controls. In vitro analyses showed that the ectopic increase of piR-823 in GC cells reduced cell growth in both cell lines in a dose-dependent manner compared to controls. In vivo experiments also evidenced a significant decrease in tumor volumes and tumor weight in nude BALB/c mice xenografted with MGC-803 cells transfected with different doses of piR-823 mimics, which strongly suggests that piR-823 could act as a tumor-suppressor piRNA in the cell by avoiding the tumor formation [64].

In 2016, Martinez et al. analyzed the RNA-sequencing (RNA-seq) libraries of 320 gastric adenocarcinomas and 38 non-malignant tissues obtained from The Cancer Genome Atlas (TCGA) Project datasets in order to evaluate the expression of small RNAs and their association with clinicopathological variables. About 156 piRNAs were significantly deregulated in GC and most of them were overexpressed in GC, suggesting a potentially important role in GC. Interestingly, 70.6% of these piRNAs were not originated from known human piRNA clusters but they were mainly derived from protein-coding sequences, which have been associated with cis- and trans-regulatory effects on protein-coding transcripts in diverse species. The authors also found that lower piR-FR222326 expression was associated with poor overall survival (OS), whereas the higher expression of five of the selected piRNAs was significantly associated with lower recurrence-free survival (RFS). These RFS-associated piRNAs were then grouped into a multi-piRNA panel to evaluate the RFS prediction level in a GC cohort. Results showed that the piRNA panel composed of piR-FR290353, piR-FR064000, and either piR-FR387750 or piR-FR157678 was able to effectively stratify GC patients into low-risk and high-risk recurrence groups, demonstrating that certain piRNAs, as well as other non-coding RNAs, can be associated with GC patient outcome [65].

Further analyses evaluated the usefulness of this piRNA panel in the RFS data of nine additional tumor types. In this regard, the panel tended to behave similarly in colon cancer, suggesting conserved importance to digestive tract malignancies. Next, the authors assessed whether DNA copy number was associated with expression changes of the five piRNAs associated with RFS in the same TCGA cohort, confirming that, effectively, the alteration in the copy number observed in piR-FR381169, piR-FR290353, and piR-FR064000 loci was significantly associated with expression alterations, suggesting genetically selected mechanisms of deregulated piRNA expression in these cases. Finally, researchers validated expression levels of this RFS-related piRNA panel in an independent cohort composed of sequencing libraries from 25 GC belonging to the Gene Expression Omnibus (GEO) dataset, whose results were subsequently compared to those found in the TCGA cohort. The results showed that the piRNA panel composed of piR-FR290353 and piR-FR387750/piR-FR157678 was still useful to accurately predict RFS in GC cases [65].

#### 5.1.2. The piRNAs as Diagnostic Markers in Gastric Cancer (GC)

A study by Cui et al. assessed the expression levels of piR-651 and piR-823 in the peripheral blood of 93 GC patients and 32 healthy volunteers, finding that both piRNAs were significantly lower in the blood of GC patients. In fact, piR-651 levels were even lower in the blood samples of patients with gastric signet ring cell carcinoma; meanwhile, the piR-823 levels were associated with cases of advanced T stage and presence of distant metastases. Interestingly, the levels of both piRNAs were found lower in the blood of postoperative patients than those from preoperative patients, which allows these piRNAs to be considered for the follow-up of patients after a determined treatment. Then, the diagnostic usefulness of these transcripts was assessed through the receiver operating characteristic (ROC) curves that showed “area under the curve“ (AUC) values of 0.841 for piR-651, 0.812 for piR-823, and 0.860 for the combination of both piRNAs, which were better than those AUC values obtained by the same group for other small RNAs (miR-106a and miR-17). In addition, comparing the positive detection rates, piR-651 and piR-823 showed to be more sensitive than serum measuring of routine tumor markers CEA and CA19-9. These robust data suggest that both piRNAs might be valuable blood biomarkers to distinguish GC patients from healthy subjects with high sensitivity and specificity [79].

Other piRNAs with a potential role as biomarkers are piR-018569, piR-004918, and piR-019308, which were evaluated in serum exosomes from 70 GC patients and 60 healthy donors, being found significantly elevated in the GC cases. In particular, piR-004918 and piR-019308 had significantly higher expression levels in the blood of patients with metastasis. The ROC curves evidenced that serum levels of piR-019308, piR-004918, and piR-018569 were able to distinguish between GC patients and healthy individuals, with AUC values of 0.820, 0.754, and 0.732, respectively. These AUC values were even better than those obtained by the routine tumor markers CEA, CA19-9, and AFP (AUC values of 0.689, 0.687, and 0.634, respectively). In fact, when piR-019308 or piR-004918 or piR-018569 were evaluated in combination with CEA and CA19-9, the AUC of each biomarker panel reached values of 0.914 (*p* < 0.0001), 0.859 (*p* < 0.0001), and 0.868 (*p* < 0.0001), respectively. Therefore, these data suggest that piR-019308, piR-004918, and piR-018569, mainly piR-019308, combined with CEA and CA19-9 can serve as reliable markers to detect GC [20].

### 5.2. Colorectal Cancer (CRC)

Colorectal cancer (CRC) is the third most frequently diagnosed cancer worldwide and the second cause of cancer deaths, with ~1.80 million new cases and ~880,000 deaths per year, respectively [77,80]. These CRC cases are mainly sporadic (70–80%) and have been associated with risk factors such as age and lifestyle without a family history of disease or genetic predisposition [81]. The evidence has demonstrated that CRC is a heterogeneous disease and its pathogenesis involves the activation of oncogenes and inactivation of tumor-suppressor genes, which are mostly the result of genetic mutations and epigenetic alterations, the latter including DNA methylation, histone modification, and non-coding RNAs (ncRNAs) [82].

#### 5.2.1. The piRNAs as Prognosis Markers and Regulators of Colorectal Cancer (CRC)

Various piRNAs have been involved in the regulation of the colorectal carcinogenic process. One of these piRNAs is piR-823, which was found significantly upregulated in the CRC tissues compared to non-tumor adjacent tissues. This higher piR-823 expression was correlated with poorly differentiated tumors [66]. In vitro experiments showed that inhibition of piR-823 resulted in suppression of cell proliferation and colony formation, cell cycle arrest in the G1 phase, and induction of cell apoptosis in CRC cell lines HCT116 and DLD-1, whereas overexpression of piR-823 promoted cell proliferation in normal colonic epithelial cell line FHC. Interestingly, piR-823 was shown to increase the transcriptional activity of HSF1 (a common transcription factor of some members of the heat shock protein (HSP) family such as HSP27, HSP60 and HSP70) by inducing the phosphorylation of HSF1 at Ser326 [66]. Another study conducted by Sabbah et al. evaluated the piR-823 expression levels in tissues and serum of CRC patients. In the case of tissue samples, the authors confirmed the overexpression of piR-823 in CRC tissues and its association with those poorly differentiated tumors. In addition, they also found that higher piR-823 levels were associated with advanced TNM stages (III–IV) [67]. Therefore, both studies suggest that piR-823 can act as a potential prognostic marker and as a promising therapeutic target for CRC [66,67]. Sabbah et al. also studied piR-823 as a potential diagnostic biomarker in CRC and their results will be shown later.

In the case of Weng et al.’s study, they performed RNA-seq experiments and subsequent validations that resulted in the identification of piR-1245 as a significantly upregulated piRNA in CRC tumors. Interestingly, these elevated piR-1245 levels significantly correlated with poor differentiation, advanced T stage, and the presence of lymph node metastasis and distant metastasis in CRC patients. Furthermore, patients with higher expression of piR-1245 also showed significantly poor overall survival (OS). Functional experiments performed in CRC cell lines (HCT116 and SW480 cells) helped to determine that piR-1245 acts as an oncogene by promoting tumorigenesis via increasing colony formation and cell proliferation. The gene expression profiling and the analysis of piRNA:mRNA base-pairing interactions found that certain tumor-suppressor genes such as ATF3, BTG1, DUSP1, FAS, NFKBIA, UPP1, SESN2, TP53INP1, and MDX1 are direct targets of piR-1245. These targets were then validated through correlation analyses between the piR-1245 expression and the expression of each candidate gene, reaffirming the likely oncogenic role of piR-1245 and proposing this piRNA as a potential independent prognostic marker in CRC [68].

Other piRNAs also evaluated in CRC cases are piR-18849, piR-19521, and piR-17724, which have been found upregulated in tumors. No associations were found between piR-17724 expression and clinicopathological features. However, the increased piR-18849 expression was associated with poorly differentiated tumors and the greater presence of metastasis in lymph nodes; meanwhile, the higher expression of piR-19521 was associated with poorly differentiated CRC tumors, suggesting the probable usefulness of piR-18849 and piR-19521 as prognostic biomarkers for CRC patients [69].

Recently, Iyer et al. conducted a systematic transcriptomic discovery based on RNA-seq followed by a validation of piRNAs using two different clinical cohorts. This study identified piR-24000 as a small RNA markedly overexpressed in CRC. Interestingly, the high expression of piR-24000 was significantly associated with moderate and poor tumor differentiation, presence of distant metastases, and advanced tumor stage (mainly stage IV) in CRC cases. Moreover, piR-24000 overexpression had a positive but not significant association with advanced nodal metastasis and with advanced tumor invasion. These data suggest that piR-24000 may constitute an oncogene in CRC that could act as a diagnostic or prognostic biomarker or as a therapeutic target in this malignancy [70].

Another interesting study was performed by Mai et al., who analyzed the expression profile of piRNAs in CRC cases using the TCGA and GEO databases. These authors found that piR-54265 expression was higher in CRC tissues than controls, showing a significant correlation with poor overall survival (OS) and progression-free survival (PFS). Functional experiments carried out in CRC cell lines (HCT116 and LoVo cells) and BALB/c nude mice demonstrated that overexpression of piR-54265 substantially increases cell proliferation and tumor growth and invasiveness and significantly reduces apoptosis compared to controls. Conversely, the piR-54265 knockdown had the reverse results on all these cell parameters in vitro and in vivo. Molecular assays showed that piR-54265 binds PIWIL2 protein to induce the formation of PIWIL2/STAT3/phosphorylated-SRC (p-SRC) complex, which promotes the STAT3 signaling pathway activation that subsequently leads to the cell proliferation, metastasis, and chemoresistance observed in CRC cells. This research group also demonstrated that PIWIL2 interacts with STAT3 through its PAZ domain. Even more interesting, the PIWIL2/STAT3/p-SRC interaction in CRC cells changed once the piR-54265 expression was also modified, although the protein levels of PIWIL2, total STAT3, and SRC remained constant. This indicates that piR-54265 might facilitate the formation of PIWIL2/STAT3/p-SRC complex, thus becoming not only a prognostic marker but also a potential therapeutic target for this cancer [71].

Although most studies evaluate the expression of piRNAs and their relationship with clinicopathological variables or their interaction with signaling pathways, other studies such as the one carried out by Chu et al. aimed to analyze the presence of single nucleotide polymorphisms (SNPs) in the sequences of certain piRNAs. These authors evaluated the expression of all known piRNAs in a cohort of CRC patients and cancer-free individuals, identifying seven common SNPs present in nine known piRNAs frequently deregulated in CRC. The results, based on an additive model, revealed that reference SNP rs11776042 in piR-015551 had a significant protective effect on the risk of developing CRC. However, this protective effect was not considered as significant after correction for multiple comparisons. Furthermore, the authors suggested that piR-015551 might be also generated from the genomic sequence of lncRNA LNC00964-3, whose expression was also significantly lower in tumors versus normal tissues; therefore, both transcripts may be involved in the development of CRC [83].

#### 5.2.2. The piRNAs as Diagnostic Markers in Colorectal Cancer (CRC)

The usefulness of piRNAs as diagnostic markers in CRC has been assessed in several studies. For instance, Sabbah et al. evaluated the serum levels of piR-823 as a non-invasive diagnostic biomarker to detect CRC cases, finding that this piRNA was significantly increased in the serum of CRC patients compared to those samples from healthy donors. The ROC curve showed an AUC value of 0.933 for piR-823 (83.3% sensitivity and 89.3% specificity) [67], which reaffirms the idea that this piRNA not only could be considered a promising prognostic marker and therapeutic target for colorectal neoplasia but also could be used as a biomarker to detect this malignancy in the future.

Another study by Vychytilova-Faltejskova et al. carried out an RNA-seq screening to determine piRNA profiles in serum samples of CRC patients (before surgery) and healthy donors. They proportionally divided the samples into different sets, based on the TNM stage, for each investigation phase: screening (144 cases and 96 controls), training (80 cases and 80 controls), and validation (179 cases and 100 controls). As the different phases of the study progressed, piR-5937 and piR-28876 were selected as transcripts enable to differentiate CRC cases (even in stage I of disease) from healthy cases with higher sensitivity and specificity than routine CRC biomarkers CEA and CA19-9. PiR-5937 and piR-28876 were found downregulated in blood samples of CRC cases compared to healthy individuals and were inversely correlated with advanced clinical stage, in a significant manner. Interestingly, when piR-5937 and piR-28876 expression was assessed in the blood of these patients 1 month after the surgical resection, the levels of both piRNAs were increased significantly. These results indicate that although the role of piR-5937 and piR-28876 in colon carcinogenesis is not well defined, their serum levels could be useful to diagnose CRC even in early stages and to potentially carry out a postoperative follow-up of patients with resected CRC [84].

Serum samples were also used to evaluate the role of piR-54265 as a diagnostic biomarker. Mai et al. found that high levels of piR-54265 observed in serum of CRC patients significantly correlated with those values observed in CRC tumor tissues. Particularly, the piR-54265 overexpression was significantly associated with advanced tumors, the presence of metastases, and poor survival in CRC cases. Since piR-54265 was involved in the chemotherapeutic response, the authors also evaluated the correlation between serum piR-54265 levels and the curative efficacy of preoperative neoadjuvant chemotherapy composed of 5-fluorouracil and oxaliplatin in 317 CRC patients. In this regard, patients with low serum piR-54265 levels had a significantly better chemotherapeutic response than those with high serum piR-54265 levels, indicating a remarkable association between serum levels of this piRNA and disease progression or control rate of chemotherapy in CRC patients. Additionally, the results of this study evidenced that serum levels of piR-54265 accurately discriminate between those subjects whose CRC is progressive from those individuals whose CRC is non-progressive, including those clinical beneficiaries of a complete response, partial response, or stable disease after neoadjuvant chemotherapy [71].

The diagnostic usefulness of piR-54265 in serum was evaluated in more depth later by analyzing the blood of 725 CRC patients, 209 cancer-free healthy controls, 1303 patients with other types of digestive cancers, and 192 patients with benign colorectal tumors. The outcomes evidenced that serum piR-54265 levels were significantly elevated only in subjects with CRC compared to individuals from other groups analyzed, with an AUC value of 0.896 (sensitivity of 85.7% and specificity of 65.1%) to recognize CRC cases. Interestingly, serum piR-54265 levels declined substantially in those patients who underwent surgery but increased again in those cases whose tumors relapsed. On the other hand, prospective case-control analysis showed that the prediagnostic serum piR-54265 levels were significantly associated with future CRC diagnosis, with odd-ratio (OR) values of 7.23, 2.80, 2.45, and 1.24 for those CRC cases diagnosed within 1, 2, 3, and >3 years, respectively. Therefore, as a conclusion, serum piR-54265 analysis was found to be more sensitive than other CRC markers evaluated in blood, constituting a promising biomarker for CRC screening, early detection, and clinical surveillance [85]. However, a recent study by Tosar et al. suggests that piR-54265 could be actually a fragment of a full-length sequence belonging to a snoRNA called SNORD57 found in the serum of CRC patients; thus, methodological considerations must be taken to reliably analyze a determined piRNA or other small ncRNAs [86].

Another interesting study by Qu et al. also performed a three-phase study to establish a serum panel of piRNAs that may have potential clinical value in CRC cases. The screening phase consisted of an RNA-seq analysis carried out in the serum samples of 10 CRC patients with TNM I-II stage, 10 CRC patients with TNM III-IV stage, and 10 healthy controls, in which 16 downregulated piRNAs were selected to be evaluated in the training phase. This second phase allowed the selection of five piRNAs (piR-001311, piR-004153, piR-017723, piR-017724, and piR-020365) as significantly repressed in CRC cases; thus, researchers constructed two diagnostic panels to compare their screening performance during the validation phase: a piRNA-based panel (panel I) and CEA-based panel (panel II), which were evaluated separately (I vs. II) and in combination (I + II) using another independent cohort of 100 CRC patients and 100 healthy controls. Results demonstrated that the piRNA-based panel was better than the CEA-based panel to detect CRC, with an AUC value of 0.867 for this panel I (sensitivity of 78.3% and specificity of 74.2%). Accordingly, Kaplan-Meier analysis showed that patients with low serum piR-017724 levels had worse survival (OS and PFS), which was consistent with the multivariate Cox regression analysis that indicates that serum piR-017724 was an independent prognostic factor for OS and PFS. Therefore, the piRNAs that constitute the mentioned serum piRNA panel (panel I), especially piR-017724, have a promising utility not only as biomarkers for CRC detection but also as prognosis predictors at the time of diagnosis [87].

Recently, Iyer et al. also evaluated the expression of piR-24000 in blood samples of CRC patients, demonstrating a strong diagnostic accuracy of this piRNA to differentiate CRC patients from normal subjects. However, when researchers subgrouped the patients into early-stage (stages I and II) and late-stage (stages III and IV) groups, the early-stage group had lower AUC, sensitivity, and specificity values compared to those obtained from the late-stage group (Table 2). These results indicate that piR-24000 may serve as a biomarker that strongly discriminates between CRC patients and control subjects [70]; however, its usefulness in early diagnosis of CRC could have a lower performance.

### 5.3. Pancreatic Cancer (PC)

Pancreatic cancer (PC) has ~458,918 new cases and causes ~432,242 deaths per year, being considered as the seventh leading cause of cancer-related deaths worldwide [80]. Histologically, pancreatic ductal adenocarcinoma (PDAC) constitutes 90% of PC cases and, due to the broad heterogeneity of genetic mutations and dense stromal environment, this neoplasm is considered one of the most chemoresistant cancers [89]. Certain risk factors have been associated with PC development, including tobacco smoking, diabetes mellitus, obesity, dietary factors, alcohol abuse, age, ethnicity, family history and genetic factors, *Helicobacter pylori* infection, non-O blood group, and chronic pancreatitis [90]. Unfortunately, PC patients seldom exhibit symptoms until an advanced stage of the disease, which induces a late diagnosis. For this reason, this malignancy evidences a 5-year survival rate of only 9%, becoming one of the most lethal malignant neoplasms in terms of prognosis [90]. Moreover, regarding diagnosis, some studies have stated that screening of large groups is not considered useful to detect PC at an early stage; therefore, newer techniques and the screening of tightly targeted groups, especially individuals with family history, are being evaluated [90]. In this regard, new early-detection markers and therapeutic targets could be useful to help to overcome the multiple troubles that PC has in relating to diagnosis and treatment.

In a similar manner to GBC, few studies have been involved in evaluating the expression profiles of piRNAs and their biological role in PC. An example is a study by Miller et al., who performed a massive analysis of cDNA ends (MACE) along with an RNA-seq analysis to characterize the complete transcriptome (mRNA, miRNAs, snoRNAs, lncRNAs, and piRNAs) in tissues from six PDAC patients and five non-tumoral controls. Among the many transcripts differentially expressed between PDAC cases and normal controls, the authors found that piR-017061, a piRNA transcribed from the sequence of snoRNA HBII-296A, was significantly downregulated in PDAC compared to normal pancreas tissues [72].

Later, Xie et al. continued investigating the implication of piR-017061 in PDAC. They confirmed that this piRNA was significantly downregulated in both PDAC samples and cell lines (PANC-1 and BxPC-3 cells) compared to controls (non-tumoral adjacent tissues and HPDE6-C7 cell line). In addition, those PDAC patients with higher piR-017061 expression levels had significantly better overall survival. In vitro experiments modulating the piR-017061 expression in PANC-1 and BxPC3 cells demonstrated that piR-017061 normally inhibits cell growth and clonogenicity and promotes pancreatic cell apoptosis. These results were validated on in vivo experiment using BALB/c nude mice xenografted with PANC-1 cells previously transfected with piR-017061 mimic, which developed smaller tumors with a greater number of apoptotic cells compared to controls. Conversely, in vivo experiments using piR-017061 inhibitors evidenced the opposite effects. Then, bioinformatics and molecular analyses revealed a direct interaction between piR-017061 and PIWIL1 to bind and subsequently degrade the mRNA of the *EFNA5* gene, which encodes an ephrin localized in the membrane of cells. Therefore, the loss of piR-017061 observed in PDAC cases results in the accumulation of EFNA5, which facilitates PDAC development. Hence, these data provided novel insights into PIWI/piRNA-mediated gene regulation in PDAC and provide background about a novel therapeutic strategy for this malignancy [73].

### 5.4. Hepatocellular Carcinoma (HCC)

Primary liver cancer is the sixth most common cancer in the world and the fourth most common cause of cancer deaths [77]. Primary liver cancer is a group of pathologically heterogeneous malignancies, including mainly hepatocellular carcinoma (HCC), as well as other less frequent neoplasms such as intrahepatic cholangiocarcinoma, mucinous cystic neoplasms, intraductal papillary biliary neoplasms, hepatoblastoma in children, angiosarcoma, etc., all of them with different underlying etiologies and carcinogenic mechanisms [77]. Hepatocellular carcinoma (HCC) constitutes ~90% of primary liver cancer, with the infections by Hepatitis-B virus (HBV) and hepatitis-C virus (HCV) the contributors of ~80% of HCC worldwide [91]. Other risk factors are aflatoxin exposure, alcohol consumption, obesity, and non-alcoholic steatohepatitis (NASH), associated with metabolic syndrome or diabetes mellitus [92].

Few studies about piRNAs in liver cancer have been performed and these studies have focused primarily on piR-Hep1 and piR-823. Law et al. performed RNA-seq in HCC cell lines (HKCI-4 and HKCI-8 cells) and immortalized hepatocyte cell line (MIHA cells), discriminating about 171 differentially expressed piRNAs in HCC cell lines. The most interesting piRNA within this set of transcripts was piR-Hep1, which not only was confirmed as upregulated in HCC cell lines but was also found highly expressed in HCC tissues compared to normal livers and non-tumoral adjacent liver tissues. Moreover, increased piR-Hep1 expression was detected in the non-malignant adjacent tissues compared to normal liver tissues. In vitro assays in HKCI-8 and MIHA cells evidenced that piR-Hep1 overexpression promotes an increment in cell viability and cell migration and invasion, accompanied by increased AKT phosphorylation. This evidence shows that piR-Hep1 may act as an oncogenic small non-coding RNA in the hepatic tissue through the activation of AKT and its associated signaling pathways. More interestingly, since cirrhosis and chronic hepatitis are often considered precancerous lesions of HCC, and the expression of piR-Hep1 in these lesions is higher than normal liver tissues, the elevated piR-Hep1 levels may indicate the risk to develop liver cancer in an individual [74].

In another study by Rizzo et al., RNA-seq analysis was also performed in a total of 55 samples composed of 14 cirrhotic nodules (CN), 9 low-grade dysplastic nodules (LGDN), 6 high-grade dysplastic nodules (HGDN), 6 early HCC (eHCC), and 20 progressed HCC (pHCC) collected from 17 patients in order to determine piRNA expression profiles along the hepatic carcinogenic progression. This study found a pattern of 125 piRNAs capable of discriminating between HCC tissues from matched CN tissues, which also showed a correlation with the presence of microvascular invasion in HCC. Additional functional and predictive bioinformatic analyses showed that these deregulated piRNAs have interesting RNA targets, most of them genes that belong to recognized signaling pathways involved in hepatocarcinogenesis and HCC progression, on which these piRNAs could exert their function. Interestingly, 24 piRNAs showed specific expression patterns in dysplastic nodules compared to cirrhotic liver and/or pHCC. These findings suggest that certain piRNAs can be used as differential diagnostic biomarkers to determine both preneoplastic and neoplastic liver disease [75]. Some of these piRNAs are indicated in Table 1.

Among those piRNAs that Rizzo et al. found gradually increased in the progression from cirrhosis to dysplastic nodules and towards HCC, piR-823 deserves to be highlighted [75] because this piRNA has been also implicated in other cancer types including gastric and colorectal cancer and may play an important role in HCC as well. In this regard, Tang et al. investigated the role of piR-823 in the activation of hepatic stellate cells (HSCs), a cell type that usually is activated after infections, long-term alcohol consumption, cholestasis, and chronic metabolic disorders, becoming the main source of myofibroblasts that synthesize the components of extracellular matrix (ECM) that results in liver fibrogenesis, cirrhosis, and, subsequently, liver carcinoma. Effectively, these authors found that piR-823 was significantly upregulated in activated HSCs from CCL4-treated C57BL/6 mice compared to those HSCs from control mice. In fact, they proved that high piR-823 levels in LX-2 cells (a human hepatic stellate cell line) induced greater proliferation and higher levels of two pro-fibrotic proteins: α-smooth muscle actin (α-SMA) and collagen I (COL1A1). Conversely, the inhibition of piR-823 by antagomiRs significantly reduced the activity of HSCs. Pull-down assays showed that piR-823 can interact with several types of proteins, such as SFPQ, PLOD1, and EIF3B. In particular, EIF3B is a translation promoter of TGF-β1, which is a well-recognized factor in the pathogenesis of liver fibrosis; therefore, the interaction between piR-823 and EIF3B can induce a higher TGF-β1 expression that leads to greater activation of HSCs to initiate liver fibrosis. In this manner, piR-823 may be also considered a promising target in the treatment of liver fibrosis and, hence, its modulation would help to avoid the development of HCC [93].

### 5.5. Gallbladder Cancer (GBC)

Gallbladder cancer (GBC) is the most frequent neoplasm of the biliary tract, representing ~80–95% of biliary tract malignancies [94]. Around the world, GBC is the 22nd most incident but 17th most deadly cancer, representing 1.7% of all cancer-related deaths and constituting the fifth most common gastrointestinal neoplasms [80,94]. The incidence of GBC varies depending on the geographic region and ethnicity, being an important cause of mortality in Japan, India, and Chile, among other countries [95]. The most important risk factor described for the GBC is gallstone disease (GSD), but some environmental and lifestyle risk factors can induce the development of gallstones and the subsequent GBC formation such as age, geographical location, female gender, ethnicity, nutritional aspects (high-fat and high-sugar diets), bacterial infections (e.g., Salmonella typhi), chronic diseases (e.g., sclerosing cholangitis, type II diabetes, metabolic syndrome, and/or dyslipidemias), parasitic infections, smoking, and alcohol consumption [96,97,98]. Unfortunately, since GBC is usually asymptomatic in the early stages and thereby its diagnosis is late, this diagnosis occurs in an advanced stage of the disease that shows an invariable course to death because all treatments become ineffective, showing an overall survival rate of only 6 months on average, with a 5-year survival rate of 5% [99]. Thus, new markers or therapeutic targets are needed to overcome these screening issues observed in GBC.

There are scarce studies that have analyzed the expression of piRNAs in GBC. The most important study was the one performed by Gu et al., who aimed to identify the piRNA signature present in blood exosomes of five cholangiocarcinoma (CCA) and four GBC patients and five healthy individuals. The authors focused on selecting those differentially expressed piRNAs with the highest fold-change values between healthy donors and patient groups (CCA + GBC), resulting in the identification of 10 upregulated piRNAs within CCA + GBC cases, including piR-2660989, piR-10506469, piR-20548188, piR-10822895, piR-23209, and piR-18044111. In particular, piR-23209 and piR-17603885 were suggested as promising biomarkers in the diagnosis of both CCA and GBC cases. On the contrary, certain piRNAs such as piR-17802142, piR-12355115, piR-4262304, piR-5114107, piR-9052713, and piR-14022777 were found downregulated in this CCA + GBC group. Interestingly, piR-12355115 showed dramatically low levels in both CCA and GBC cases, suggesting that its downregulation can be used as a common biomarker for these two diseases.

Later, some of these piRNAs were evaluated in a validation cohort composed of blood samples from 50 healthy individuals, 40 CCA patients, and 25 GBC patients. Results showed that piR-10506469 expression was significantly increased in the plasma exosomes of CCA and GBC patients, confirming the results of RNA-seq. Additionally, piR-20548188 and piR-14090389 were found significantly upregulated only in the exosomes of CCA patients. In particular, the levels of piR-14090389 were increased as the malignant grade increased of GBC cases.

The most interesting results of this study were found when researchers compared the expression of certain piRNAs in the blood of patients before surgery and 1 week after the surgery. For instance, the expression levels of piR-10506469 and piR-20548188 were found considerably diminished in plasma of CAA and GBC cases 1 week after surgery compared to individuals before surgery, demonstrating that these piRNAs constitute potential circulating diagnostic markers to differentiate CCA and GBC cases from healthy individuals and to carry out post-treatment follow-up in patients with gallbladder diseases. On the other hand, the expressions of piR-4333713 and piR-14090389 were found significantly repressed in the respective blood samples of GBC and CCA obtained 1 week after surgeries compared to those samples before surgery, suggesting that these two piRNAs can be used to discriminate the progression of a healthy subject to CCA and then to GBC, becoming potential biomarkers for the diagnosis of gallbladder preneoplastic and neoplastic diseases [88].

Undoubtedly, GBC is a disease with a large and unknown field to investigate the expression of piRNAs and their role in the development of this neoplasm. Table 2 reviews the different piRNAs that have been studied as diagnostic and prognostic biomarkers in the blood of patients with GI cancers.

## 6. Experimental and Technical Considerations for RNA-Involving Studies

The best manner to obtain reliable and reproducible results through the RNA-seq platform is following certain basic requirements regarding two parameters: (1) the experimental design and (2) the suitability and quality of the samples analyzed. Among the parameters of a good experimental design, it is important to have an acceptable sample number between the groups to be contrasted in order to strengthen the comparative statistical analyses that allow the discovery of differentially expressed RNAs. In this regard, the authors have not agreed to define what is the ideal minimum number within a group to make reliable comparisons and they always leave it to the researchers’ discretion concerning what they intend to do in the study. However, no doubt, using three or four tumor tissues versus three or four non-tumor tissues adjacent to the tumor from the same patients does not seem to be a strategy that allows reaching reliable and robust results when performing the post-sequencing bioinformatics’ analyses. Unfortunately, some of the studies cited in this review begin with a piRNA discovery process that uses very few sequenced tissue samples, which could lead us to suppose the existence of certain biases in the data associated with an inappropriate experimental design. Therefore, it is suggested to increase the sample size of each group to be compared in future RNA-seq studies or using databases to increase the statistical power of the research, as did some research groups also cited in this article.

Among the parameters of the suitability and quality of the samples analyzed, those studies using RNA-seq should consider evaluating samples undergoing the best possible preservation protocols. In addition, the authors should even report the preanalytical quality values of these samples before performing RNA-seq, such as RNA integrity number (RIN) values, A260/A280 ratios, and A260/A230 ratios, among other parameters to validate their results before the scientific community. In the case of blood samples used to assess exosomal or free circulating piRNAs, most studies did not perform or report the evaluation of the RNA stability in plasma over time at different temperatures or the stability of these molecules between the successive freezing and thawing steps. Fortunately, there are a few good examples. For instance, the study performed by Qu et al. was able to explore the feasibility of certain circulating piRNAs as biomarkers for CRC diagnosis using serum samples submitted to room-temperature incubation (for 4, 8, and 24 h) and repetitive freeze-thaw cycles after incubation at −80 °C for 1, 2, and 3 months. Despite serum samples being exposed to harsh conditions, the Ct values of specific piRNAs showed no significant variation, demonstrating that piRNAs were sufficiently stable in serum and, hence, they could be used for the routine processing of clinical samples. Similar analyses should be encouraged in future studies assessing either free-circulating or exosomal piRNA levels in serum samples.

Regarding the tissues used to perform RNA-seq analysis, some studies used formalin-fixed and paraffin-embedded (FFPE) tissue samples to carry out their discovery stage, instead of using fresh frozen tissues. Other studies could be using FFPE tissues in the validation stages. However, in all cases, the best tissue samples to be used in RNA studies are fresh frozen tissues because this is the sample preservation technique that most likely prevents the degradation of RNA molecules by the action of nucleases performed at room temperature. Therefore, the protocols for collecting, biomolecule extraction, and pre-analytical procedures of these fresh frozen tissues should be closely controlled to guarantee reliable, comparable, and accurate results.

Finally, another important issue to be considered in piRNA-related studies is the wise and careful use of databases and bioinformatics’ tools. This topic has been discussed since the massification of high-performance platforms and the rise of in silico tools since a careless analysis of sequences may lead to confusion that could not only impact the methodology of a study but also the clinical approach of this research line. For example, this review mentioned that serum detection of certain piRNAs allows excellent discrimination between GI cancer patients and healthy controls. However, recent studies have shown that most of the mammalian somatic piRNAs reported so far in databases and in silico tools are actually fragments of other, longer ncRNAs erroneously entered. In fact, for avoiding this misunderstanding, Tosar et al. introduced the term miscellaneous-piRNA (m-piRNA) to distinguish between a canonical piRNA and other small RNAs circumstantially associated with PIWI proteins in somatic cells [86].

Therefore, researchers who follow this research line must be cautious and rigorous in the way they execute bioinformatics and pre-analytical and analytical analyses and must endeavor to fully report their results to the community so that we can all draw the correct conclusions.

### 6.1. The piRNAs as Potential Biomarkers in GI Cancers

In terms of diagnosis and prognosis, some of these cancer-specific piRNAs have been demonstrated to be detected in the tissues and plasma of cancer patients with interesting results. Beyond detection and quantification of piRNA expression in these clinical samples, the application of piRNAs as biomarkers in daily practice can be challenging given their nature as RNA molecules. The desirable markers should be stable and easily detectable in plasma or other body fluids to allow non-invasive diagnosis; therefore, the analysis of piRNAs contained in exosomes, microvesicles, apoptotic bodies, and apoptotic microparticles as biomarkers must continue to be encouraged because these membranous particles not only are released by tumor cells but also can prevent RNA degradation due to RNases [100,101,102]. In this regard, the blood-circulating piRNAs have emerged as a potential tool in the screening, selection, and follow-up of GI cancer patients to provide personalized treatments [102]. These piRNAs can be released from tumor cells either in a cell-free form or within microvesicles, such as exosomes. The main advantages of analyzing blood-circulating piRNAs are the non-invasive sampling, a good correlation with tumor size, better detection sensitivity, and good stability in the bloodstream (especially those piRNAs transported within exosomes) compared to other cell-free nucleic acids [100,101,102]. Interestingly, some piRNAs are more accurate, either alone or as a piRNA panel, compared to conventional GI tumor markers such as CEA and CA19-9 in the different cohorts analyzed. However, more exhaustive clinical validations are needed to define whether piRNAs are worse or better than other clinical, genetic, or epigenetic markers.

Despite that blood-circulating piRNAs could only be used as complementary markers until now, in the future, these piRNAs might be important to select the drug protocol and to perform the follow-up of cancer patients to evaluate the effectiveness of drug protocols. However, as explained before, some suggestions should be considered in the studies that propose novel blood-circulating biomarkers: (1) The sample size should be greater at the different validation stages and (2) the results of the preanalytical analyses (e.g., RNA quality indexes, stability over time at different temperatures, stability before successive freezing/thawing of samples, etc.) should be reported when corresponding.

### 6.2. The piRNAs as Potential Targeted Therapy in GI Cancers

In terms of therapy, future studies must be more focused on understanding the molecular and functional implications of piRNAs and/or PIWI proteins, particularly their specific regulation on DNA, mRNA, miRNA, or protein activity. This knowledge might be useful to develop drugs against piRNAs or PIWI proteins, which may be less toxic than conventional chemotherapy and protein-specific inhibitory drugs. Another potential therapeutic benefit could be obtained through RNA-based therapeutic strategies, such as small-interfering RNAs (siRNAs), microRNAs (miRNAs), antisense oligonucleotides (ASOs), aptamers, synthetic mRNAs, and other molecules [103], which could be designed specifically to act as determined piRNAs to target another molecule, against a specific piRNA or ribonucleoprotein complexes. These RNA-based therapeutics have not yet been widely used in clinical trials but they offer three major advantages over other traditional therapy such as chemotherapy, small-molecule inhibitors, or antibodies: (1) Once delivered to a specific cell type or tissue has been devised, every disease-promoting gene in that cell type can likely be targeted; (2) RNA-based therapeutics can selectively target single genes and can be readily engineered to keep away from off-target genes, whereas small-molecule inhibitors often hit multiple targets and have unknown off-target binding; and (3) unlike static small-molecule inhibitors and antibodies, RNA therapeutics can “pharmacoevolve” their sequence at the same pace as disease [103]. Therefore, RNA-based therapeutics have considerable potential to treat undruggable human diseases but these strategies have to solve two main problems: the delivery and the toxicity of their chemical vehicles [104,105].

Despite these potential tools being promising, the main challenges are the safe delivery and the specificity within the cells mainly at a genomic level, especially on in vivo models. Genome editing by clustered, regulatory interspaced, short palindromic repeats- associated endonuclease 9 (CRISPR-Cas9) might be considered a solution for these specificity limitations by inducing total or partial modulation of piRNA clusters and the subsequent phenotypical changes in cancer cells; however, at present, there are not many CRISPR-Cas9 developments aimed at modifying the expression of piRNAs. In addition, the use of CRISPR-Cas9 might also have some challenges because piRNAs are small RNA sequences and, as indicated by Martinez et al. for GC [65], most of the expressed piRNAs do not originate from known human piRNA clusters but, instead, about 70.9% of these piRNAs were derived from protein-coding sequences, which could hinder the results expected through this strategy.

Therefore, RNA-based therapeutics have considerable potential to treat undruggable human diseases but these strategies have to solve two main problems: (1) the delivery of these molecules into the specific tumor cells and avoiding kidney clearance or liver absorption, and (2) the toxicity of their chemical vehicles that involves a higher immune response in mice, which is mainly seen as tissue damage in some organs, such as liver, kidneys, and brain, in these animals [104,105].

## 7. Concluding Remarks

Late diagnosis, metastasis development, and drug resistance seem to be the major complications of GI cancers and remain significant challenges for reaching the optimal cancer remission outcomes. However, emerging data from high-throughput technologies, such as RNA-seq platforms, have provided valuable insight into the molecular classification of GI cancers and intracellular pathways involved in both the initiation and the appearance of aggressive features of these neoplasms, allowing the potential development of novel treatments and diagnostic tools for these types of malignancies.

The data obtained from these high-throughput platforms not only focuses on the DNA genomic abnormalities but also on epigenetic deregulations including changes in DNA or histone methylation patterns or alteration in the expression of ncRNAs (i.e., lncRNAs, circRNAs, siRNAs, miRNAs, and piRNAs). In this review, piRNAs were described as small ncRNAs that have garnered attention in many fields in a bid to understand various physiological and pathological cell processes, such as carcinogenesis, where marked deregulation in the expression pattern of these transcripts contributes to the onset and progression of malignancies.

The most important advance to assess the piRNA patterns in cancer has been the introduction and popularization of RNA-Seq technology, which has acted as a standard tool for transcriptomic studies because this platform can be carried out on an increasing number of samples and can remove more efficiently the barriers to detecting all the existing forms of RNA molecules. In other words, RNA-Seq analyses allow the detection of several thousand uncharacterized ncRNAs present in the whole tissue or even in specific cell types inside that tissue. Other methodologies have been recently developed to analyze the interaction between these ncRNA and DNA, protein, or other RNAs to elucidate their molecular action within the cells and thereby explain the roles these transcripts play either in physiological or pathological processes. For instance, ChIRP and CHART methods, which use complementary oligonucleotides to pull down chromatin-related ncRNAs, can determine the chromatin-binding sites for each type of ncRNAs such as the case of piRNAs. Alternatively, RNA immunoprecipitation sequencing (RIP-Seq) and photoactivatable ribonucleoside-enhanced crosslinking and immunoprecipitation (PAR-CLIP) complement the study of RNA-protein interactions. These techniques have been used more and more for exploring the mechanisms that govern ncRNA-chromatin interactions, as demonstrated by some articles that have used some of these assays to determine a gene or protein target that interacts with a specific piRNA or PIWI protein to induce certain phenotypes in cancer cells. In this regard, several studies cited in this article have focused on the discovery and validation of differentially expressed piRNAs in GI cancers but only a few studies have deepened the evaluation of the molecular and functional roles of these transcripts, which are the less known features of piRNAs. The knowledge about the specific piRNA expression patterns in tissues or cells, and about their corresponding molecular and functional roles in these tissues or cells, is useful to develop accurate biomarkers for diagnosis or therapeutic targets for GI cancers in the future.

The existence of thousands of small RNA molecules, such as piRNAs, and the fact that these molecules are capable of participating in various physiological and pathological cellular regulation processes have important implications to develop an updated definition of the concept of cancer. This knowledge about ncRNA, in general, would help us to see the origins and progression of this disease with different eyes and open the spectrum for new treatments in the future. However, although the roles of piRNAs in different neoplasms have just begun to be revealed thanks to the rapid development of high-throughput detection technologies and supplementary molecular techniques, more advances are needed to implement this knowledge in clinical practice, including: first, the development of better and more specific biochemical or pharmacological molecules (e.g., oligonucleotides or chemical inhibitors) that allow modifying the expression and activity of a certain piRNA in the tumor, and second, the development of efficient delivery methods that allow these molecules to be preserved from the enzymatic activity and immune system during their journey to the tumor, to reach a specific tumor cell and perform their function there. An obvious requirement to reach these development stages is to have a more complete understanding of the functions and mechanisms used by piRNAs in both physiological and pathological conditions. Therefore, at the present, not only is it important to have a description of the expression level of these piRNAs in the different conditions but also, more importantly, to establish more precisely what are the functions of these piRNAs within each type of tumor cell.

## Figures and Tables

**Table 1:** The role of piRNAs in GI cancers.

GI Cancer Type	piRNA/Refs	Expression in Cancer	Experimental Design	Summary of Findings
Gastric cancer (GC)	piR-651 [63]	Up	*Tissues samples* GC vs. NAT *In vitro* MGC-803 and SGC-7901 cells transfected with mimic.	The piR-651 overexpression is associated with a greater TNM stage. The piR-651 induces cell growth and proliferation by inhibiting the cell cycle arrest at the G2/M phase.
	piR-823 [64]	Down	*Tissues samples* GC vs. NAT *In vitro* MGC-803 and SGC-7901 cells transfected with piR-823. *In vivo* BALB/c mice xenografted with MGC-803 after mimic transfection.	The piR-823 showed no association with clinicopathological features. The piR-823 inhibits cancer cell proliferation in vivo. The piR-823 decreases tumor growth and tumor weight in vivo.
	piR-FR222326 [65]	Down	*Dataset* GC vs. NT (from *TCGA*)	Lower piR-FR222326 expression was associated with poor overall survival (OS)
	piR-FR290353 piR-FR064000 piR-FR387750 piR-FR157678 [65]	Up	*Dataset* GC vs. NT (from *TCGA*) GC *TCGA* + GC *GEO*	These piRNAs (except piR-FR064000) were associated with poor RFS and could stratify GC cases into low and high risk of recurrence groups. After comparison between TCGA and Omnibus, piR-FR290353 and piR-FR387750 or piR-FR157678 could predict survival (RFS).
Colorectal cancer (CRC)	piR-823 [66,67]	Up	*Tissues samples* GC vs. NAT *In vitro* HCT116 and DLD-1 cells transfected with inhibitor. FHC cells transfected with mimic.	*Tissues samples* High piR-823 expression correlated with poorly differentiated tumors. *In vitro* The piR-823 upregulation activates the HSF1/HSPs’ pathway to induce cell proliferation and colony formation.
	piR-1245 [68]	Up	*Tissues samples* CRC vs. NT (for RNA-seq) *Dataset* CRC tissues from TCGA (*in silico* validation) *Additional tissues samples* CRC vs. NT tissues (as validation cohort) *In vitro* HCT116 and SW480 cells transfected with mimic/inhibitor	*Tissues samples* The piR-1245 overexpression was associated with poor differentiation, advanced TNM stage, lymph node metastasis, distant metastasis, and poor survival (OS). *In vitro* The piR-1245 overexpression increases cell proliferation and invasiveness and reduces apoptosis by directly targeting mRNA of tumor-suppressor genes such as *ATF3*, *BTG1*, *DUSP1*, *FAS*, *NFKBIA*, *UPP1*, *SESN2*, *TP53INP1,* and *MDX1*.
	piR-18849 piR-19521 piR-17724 [69]	Up (all of them)	*Screening tissue samples* CRC vs. NAT (by RNA-seq) *Validation in tissue samples* CRC vs. NAT	The piR-18849 was associated with poorly differentiated CRC and lymph node metastasis. The piR-19521 was associated with poorly differentiated CRC.
	piR-24000 [70]	Up	*Screening tissue samples* CRC vs. NAT (by RNA-seq) *Validation in tissue samples* CRC vs. NAT	The piR-24000 was associated with moderate and poor tumor differentiation, presence of distant metastases, and advanced tumor stage (mainly stage IV) in CRC cases. The piR-24000 had a not-significant association with advanced nodal metastasis and advanced tumor invasion.
	piR-54265 [71]	Up	*Dataset* CRC *TCGA* + CRC *GEO* (*in silico* discovery) *Validation tissues samples* CRC vs. NAT Tissues *In vitro* HCT116 and LoVo cells transfected with piR-54265 mimic/inhibitor. *In vivo* BALB/c mice xenografted with parental or resistant HCT116 and LoVo cells transfected with piR-54265 mimic/inhibitor.	*Tissues samples* The piR-54265 upregulation was directly associated to advanced TNM stage and poor survival (OS and PFS). *In vitro and in vivo* The piR-54265 upregulation increased cell proliferation, tumor growth and invasiveness, and reduced apoptosis. The piR-54265 upregulation was shown to increase resistance to 5-FU and OXA. The piR-54265 could bind PIWIL2 protein to form PIWIL2/STAT3/p-SRC complex, which activates STAT3 signaling to promote proliferation, metastasis, and chemoresistance in CRC cells.
Pancreas cancer (PC)	piR-017061 [72,73]	Down	*Screening tissue samples* PDAC vs. NT (by RNA-seq) *Validation tissue samples* PDAC vs. NT tissues PANC-1 and BxPC-3 cells vs. HPDE-C7 cells *In vitro* PANC-1 cells transfected with piR-017061 mimic. BxPC3 cells transfected with piR-017061 inhibitor. *In vivo* BALB/c mice xenografted with PANC-1 cells transfected with piR-017061 mimic.	*Tissue samples* The piR-017061 expression level had significantly higher overall survival. *In vitro and in vivo* The piR-017061 downregulation induces greater tumor growth and lower cell apoptosis. The piR-017061 was described to bind PIWIL1 for acting on the mRNA of the *EFNA5* gene to promote the development of PC.
Liver cancer	piR-Hep1 [74]	Up	*Screening cell lines* HKCI-4 and HKCI-8 cells vs. MIHA cells (by RNA-seq) *Validation tissue samples* HCC vs. NAT HCC vs. NT NAT vs. NT *In vitro* HKC-8 cells transfected with piR-Hep1 inhibitor. MIHA cells transfected with piR-Hep1 mimic.	The piR-Hep1 was found overexpressed in HCC cases and tissues of precancerous lesions of HCC (cirrhosis or chronic hepatitis) than normal liver tissues. Therefore, piR-Hep1 may be an early indicator of risk for cancer development. The piR-Hep1 was shown to increase cell viability, motility, and invasiveness, accompanied by higher p-AKT levels.
	piR-LLi-24894 [75]	Down	*Screening Tissue samples* CN vs. LGDN vs. HGDN vs. eHCC vs. pHCC (by RNA-seq)	The piR-LLi-24894 was highly expressed only in LGDN.
	piR-13306 [75]	Up (in pHCC)	*Screening Tissue samples* CN vs. LGDN vs. HGDN vs. eHCC vs. pHCC (by RNA-seq)	The piR-13306 was involved in the complete hepatic carcinogenic process, except in eHCC.

Abbreviations: NAT: non-tumoral tissue adjacent to tumor (specimens specified as non-tumor tissue from the same cancer patient); NT: non-tumoral tissue (non-tumor tissues not necessarily adjacent to the tumor and that may come from healthy subjects); OS: overall survival; RFS: recurrence-free survival; PFS: progression-free survival; CN: cirrhotic nodules; LGDN: low-grade dysplastic nodules; HGDN: high-grade dysplastic nodules; eHCC: early HCC; pHCC: progressed or advanced HCC.

**Table 2:** The piRNAs as blood biomarkers in GI cancers.

GI Cancer	piRNAs/Refs	Expression in Blood of GI Cancer Patients	Summary of Findings
Gastric cancer (GC)	piR-651 [79]	Down	The piR-651 was higher in gastric adenocarcinoma than gastric signet ring cell carcinoma. The piR-651 was lower in postoperative than preoperative patients. AUC value of 0.841 The piR-651 was found to be more sensitive than CEA and CA19-9.
	piR-823 [79]	Down	The piR-823 was lower in postoperative patients than in preoperative patients. The piR-823 was higher in patients with advanced T stage and distant metastases. AUC of 0.812 The piR-823 was found to be more sensitive than CEA and CA19-9.
	piR-018569 piR-004918 piR-019308 [20]	Up	The piR-018569 had an AUC of 0.732 The piR-004918 correlated with the presence of metastases. The piR-004918 had an AUC of 0.754 The piR-019308 correlated with the presence of metastases. The piR-019308 had an AUC of 0.820 This piRNA panel showed better results than tumor markers CEA, CA19-9, and AFP to diagnose GC.
Colorectal cancer (CRC)	piR-823 [67]	Up	Higher levels in III and IV tumor stages. Higher levels in poorly differentiated tumors. AUC of 0.933 (83.3% sensitivity and 89.3% specificity).
	piR-5937 piR-28876 [84]	Down	These piRNAs could differentiate CRC cases even from stage I. Both piRNAs had higher sensitivity and specificity than CEA and CA19-9. The levels of both piRNAs were restored after surgical resection.
	piR-54265 [71,85]	Up	The piR-54265 may serve as a predictor marker of metastasis and poor survival. The piR-54265 may serve as a predictor marker for response to chemotherapy. AUC values of 0.896 (85.7% sensitivity and 65.1% specificity)
	piR-001311 piR-004153 piR-017723 piR-017724 piR-020365 [87]	Down	This piRNA’s panel could detect CRC better than CEA. Low piR-017724 levels found in patients with poor survival (OS and PFS) The piRNA panel AUC value of 0.867 (78.3% sensitivity and 74.2% specificity).
	piR-24000 [70]	Up	The piR-24000 could significantly differentiate CRC from normal subjects, with an AUC value of 0.8175 (Sensitivity = 93.1% and Specificity = 68.97%,) Early-stage group (stages I and II) had an AUC value of 0.7796 (Sensitivity = 87.88% and Specificity = 66.67%). Late-stage group (stages III and IV) had an AUC value of 0.8405 (Sensitivity = 96.3% and Specificity = 70.37%)
Gallbladder cancer (GBC)	piR-10506469 [88]	Up	The piR-10506469 was upregulated in exosomes of CCA and GBC patients. This piRNA was associated with surgical resection of CCA and GBC.

Abbreviations: AUC: “area under the curve” value; CCA: cholangiocarcinoma; OS: overall survival; PFS: progression-free survival.

## References

[1] Arnold M., Abnet C.C., Neale R.E., Vignat J., Giovannucci E.L., McGlynn K.A., Bray F. (2020). Global Burden of 5 Major Types of Gastrointestinal Cancer. Gastroenterology.

[2] Shahjehan F., Kamatham S., Kasi P.M. (2019). Role of Circulating Tumor DNA in Gastrointestinal Cancers: Update From Abstracts and Sessions at ASCO 2018. Front. Oncol..

[3] Mereiter S., Balmaña M., Gomes J., Magalhães A., Reis C.A. (2016). Glycomic Approaches for the Discovery of Targets in Gastrointestinal Cancer. Front. Oncol..

[4] Letelier P., Riquelme I., Hernández A.H., Guzmán N., Farías J.G., Roa J.C. (2016). Circulating MicroRNAs as Biomarkers in Biliary Tract Cancers. Int. J. Mol. Sci..

[5] Riquelme I., Letelier P., Riffo-Campos A.L., Brebi P., Roa J.C. (2016). Emerging Role of miRNAs in the Drug Resistance of Gastric Cancer. Int. J. Mol. Sci..

[6] Acharya A., Markar S.R., Matar M., Ni M., Hanna G.B. (2017). Use of Tumor Markers in Gastrointestinal Cancers: Surgeon Perceptions and Cost-Benefit Trade-Off Analysis. Ann. Surg. Oncol..

[7] Zhang L., Miao R., Zhang X., Chen W., Zhou Y., Wang R., Zhang R., Pang Q., Xu X., Liu C. (2015). Exploring the diagnosis markers for gallbladder cancer based on clinical data. Front. Med..

[8] Catalano V., Labianca R., Beretta G.D., Gatta G., de Braud F., van Cutsem E. (2009). Gastric cancer. Crit. Rev. Oncol. Hematol..

[9] Esteller M. (2011). Non-coding RNAs in human disease. Nat. Rev. Genet..

[10] Ferreira H.J., Esteller M. (2018). Non-coding RNAs, epigenetics, and cancer: Tying it all together. Cancer Metastasis Rev..

[11] Zhang P., Wu W., Chen Q., Chen M. (2019). Non-Coding RNAs and their Integrated Networks. J. Integr. Bioinform..

[12] Grillone K., Riillo C., Scionti F., Rocca R., Tradigo G., Guzzi P.H., Alcaro S., Di Martino M.T., Tagliaferri P., Tassone P. (2020). Non-coding RNAs in cancer: Platforms and strategies for investigating the genomic “dark matter”. J. Exp. Clin. Cancer Res..

[13] Anastasiadou E., Jacob L.S., Slack F.J. (2017). Non-coding RNA networks in cancer. Nat. Rev. Cancer.

[14] Aravin A.A., Naumova N.M., Tulin A.V., Vagin V.V., Rozovsky Y.M., Gvozdev V.A. (2001). Double-stranded RNA-mediated silencing of genomic tandem repeats and transposable elements in the *D. melanogaster* germline. Curr. Biol..

[15] Pillai R.S., Chuma S. (2012). piRNAs and their involvement in male germline development in mice. Dev. Growth Differ..

[16] Han Y.N., Li Y., Xia S.Q., Zhang Y.Y., Zheng J.H., Li W. (2017). PIWI Proteins and PIWI-Interacting RNA: Emerging Roles in Cancer. Cell. Physiol. Biochem..

[17] Wu X., Pan Y., Fang Y., Zhang J., Xie M., Yang F., Yu T., Ma P., Li W., Shu Y. (2020). The Biogenesis and Functions of piRNAs in Human Diseases. Mol. Ther. Nucleic Acids.

[18] Rayford K.J., Cooley A., Rumph J.T., Arun A., Rachakonda G., Villalta F., Lima M.F., Pratap S., Misra S., Nde P.N. (2021). Pirnas as modulators of disease pathogenesis. Int. J. Mol. Sci..

[19] Chalbatani G.M., Dana H., Memari F., Gharagozlou E., Ashjaei S., Kheirandish P., Marmari V., Mahmoudzadeh H., Mozayani F., Maleki A.R. (2019). Biological Function and Molecular Mechanism of piRNA in Cancer.

[20] Ge L., Zhang N., Li D., Wu Y., Wang H., Wang J. (2020). Circulating exosomal small RNAs are promising non-invasive diagnostic biomarkers for gastric cancer. J. Cell. Mol. Med..

[21] Krishnan P., Damaraju S. (2018). The Challenges and Opportunities in the Clinical Application of Noncoding RNAs: The Road Map for miRNAs and piRNAs in Cancer Diagnostics and Prognostics. Int. J. Genom..

[22] Kim V.N., Han J., Siomi M.C. (2009). Biogenesis of small RNAs in animals. Nat. Rev. Mol. Cell Biol..

[23] Liu P., Dong Y., Gu J., Puthiyakunnon S., Wu Y., Chen X.-G. (2016). Developmental piRNA profiles of the invasive vector mosquito Aedes albopictus. Parasites Vectors.

[24] Peters L., Meister G. (2007). Argonaute proteins: Mediators of RNA silencing. Mol. Cell.

[25] Iwasaki Y.W., Siomi M.C., Siomi H. (2015). PIWI-interacting RNA: Its biogenesis and functions. Annu. Rev. Biochem..

[26] Vagin V.V., Sigova A., Li C., Seitz H., Gvozdev V., Zamore P.D. (2006). A distinct small RNA pathway silences selfish genetic elements in the germline. Science.

[27] Siomi M.C., Sato K., Pezic D., Aravin A.A. (2011). PIWI-interacting small RNAs: The vanguard of genome defence. Nat. Rev. Mol. Cell Biol..

[28] Czech B., Hannon G.J. (2016). One Loop to Rule Them All: The Ping-Pong Cycle and piRNA-Guided Silencing. Trends Biochem. Sci..

[29] Zimber U., Adldinger H.K., Lenoir G.M., Vuillaume M., Knebel-Doeberitz M.V., Laux G., Desgranges C., Wittmann P., Freese U.K., Schneider U. (1986). Geographical prevalence of two types of Epstein-Barr virus. Virology.

[30] Andersen P.R., Tirian L., Vunjak M., Brennecke J. (2017). A heterochromatin-dependent transcription machinery drives piRNA expression. Nature.

[31] Han B.W., Zamore P.D. (2014). PiRNAs. Curr. Biol..

[32] Dennis C., Zanni V., Brasset E., Eymery A., Zhang L., Mteirek R., Jensen S., Rong Y.S., Vaury C. (2013). “Dot COM”, a Nuclear Transit Center for the Primary piRNA Pathway in Drosophila. PLoS ONE.

[33] Murota Y., Ishizu H., Nakagawa S., Iwasaki Y.W., Shibata S., Kamatani M.K., Saito K., Okano H., Siomi H., Siomi M.C. (2014). Yb Integrates piRNA Intermediates and Processing Factors into Perinuclear Bodies to Enhance piRISC Assembly. Cell Rep..

[34] Ipsaro J.J., Haase A.D., Knott S.R., Joshua-Tor L., Hannon G.J. (2012). The structural biochemistry of Zucchini implicates it as a nuclease in piRNA biogenesis. Nature.

[35] Nishimasu H., Ishizu H., Saito K., Fukuhara S., Kamatani M.K., Bonnefond L., Matsumoto N., Nishizawa T., Nakanaga K., Aoki J. (2012). Structure and function of Zucchini endoribonuclease in piRNA biogenesis. Nature.

[36] Ross R.J., Weiner M.M., Lin H. (2014). PIWI proteins and PIWI-interacting RNAs in the soma. Nature.

[37] Izumi N., Shoji K., Sakaguchi Y., Honda S., Kirino Y., Suzuki T., Katsuma S., Tomari Y. (2016). Identification and functional analysis of the pre-piRNA 3′ Trimmer in silkworms. Cell.

[38] Han B.W., Wang W., Li C., Weng Z., Zamore P.D. (2015). PiRNA-guided transposon cleavage initiates Zucchini-dependent, phased piRNA production. Science.

[39] Zhang Y., Liu W., Li R., Gu J., Wu P., Peng C., Ma J., Wu L., Yu Y., Huang Y. (2018). Structural insights into the sequence-specific recognition of Piwi by *Drosophila* Papi. Proc. Natl. Acad. Sci. USA.

[40] Guo B., Li D., Du L., Zhu X. (2020). piRNAs: Biogenesis and their potential roles in cancer. Cancer Metastasis Rev..

[41] Webster A., Li S., Hur J.K., Wachsmuth M., Bois J.S., Perkins E.M., Patel D.J., Aravin A.A. (2015). Aub and Ago3 are recruited to nuage through two mechanisms to form a ping-pong complex assembled by Krimper. Mol. Cell.

[42] Brennecke J., Aravin A.A., Stark A., Dus M., Kellis M., Sachidanandam R., Hannon G.J. (2007). Discrete small RNA-generating loci as master regulators of transposon activity in Drosophila. Cell.

[43] Gunawardane L.S., Saito K., Nishida K.M., Miyoshi K., Kawamura Y., Nagami T., Siomi H., Siomi M.C. (2007). A slicer-mediated mechanism for repeat-associated siRNA 5′ end formation in Drosophila. Science.

[44] Yu Y., Gu J., Jin Y., Luo Y., Preall J.B., Ma J., Czech B., Hannon G.J. (2015). Panoramix enforces piRNA-dependent cotranscriptional silencing. Science.

[45] Nishida K.M., Sakakibara K., Sumiyoshi T., Yamazaki H., Mannen T., Kawamura T., Kodama T., Siomi M.C. (2020). Siwi levels reversibly regulate secondary piRISC biogenesis by affecting Ago3 body morphology in Bombyx mori. EMBO J..

[46] Muñoz-López M., García-Pérez J.L. (2010). DNA Transposons: Nature and Applications in Genomics. Curr. Genom..

[47] Cordaux R., Batzer M.A. (2009). The impact of retrotransposons on human genome evolution. Nat. Rev. Genet..

[48] Anwar S.L., Wulaningsih W., Lehmann U. (2017). Transposable Elements in Human Cancer: Causes and Consequences of Deregulation. Int. J. Mol. Sci..

[49] Lin X., Xia Y., Hu D., Mao Q., Yu Z., Zhang H.H., Li C., Chen G., Liu F., Zhu W. (2019). Transcriptome-wide piRNA profiling in human gastric cancer. Oncol. Rep..

[50] Tóth K.F., Pezic D., Stuwe E., Webster A. (2016). The piRNA Pathway Guards the Germline Genome against Transposable Elements. Adv. Exp. Med. Biol..

[51] Wang C., Lin H. (2021). Roles of piRNAs in transposon and pseudogene regulation of germline mRNAs and lncRNAs. Genome Biol..

[52] Post C., Clark J.P., Sytnikova Y.A., Chirn G.W., Lau N.C. (2014). The capacity of target silencing by *Drosophila* PIWI and piRNAs. RNA.

[53] Kuramochi-Miyagawa S., Watanabe T., Gotoh K., Totoki Y., Toyoda A., Ikawa M., Asada N., Kojima K., Yamaguchi Y., Ijiri T.W. (2008). DNA methylation of retrotransposon genes is regulated by Piwi family members MILI and MIWI2 in murine fetal testes. Genes Dev..

[54] Liu Y., Dou M., Song X., Dong Y., Liu S., Liu H., Tao J., Li W., Yin X., Xu W. (2019). The emerging role of the piRNA/piwi complex in cancer. Mol. Cancer.

[55] Chen P., Luo Y., Aravin A.A. (2021). RDC complex executes a dynamic piRNA program during *Drosophila* spermatogenesis to safeguard male fertility. PLoS Genet..

[56] Jing Z., Xi Y., Yin J., Shuwen H. (2021). Biological roles of piRNAs in colorectal cancer. Gene.

[57] Goh W.S.S., Falciatori I., Tam O.H., Burgess R., Meikar O., Kotaja N., Hammell M., Hannon G.J. (2015). piRNA-directed cleavage of meiotic transcripts regulates spermatogenesis. Genes Dev..

[58] Ng K.W., Anderson C., Marshall E.A., Minatel B.C., Enfield K.S.S., Saprunoff H.L., Lam W.L., Martinez V.D. (2016). Piwi-interacting RNAs in cancer: Emerging functions and clinical utility Kevin. Mol. Cancer.

[59] Rouget C., Papin C., Boureux A., Meunier A.C., Franco B., Robine N., Lai E.C., Pelisson A., Simonelig M. (2010). Maternal mRNA deadenylation and decay by the piRNA pathway in the early *Drosophila* embryo. Nature.

[60] Balmeh N., Mahmoudi S., Karabedianhajiabadi A. (2021). piRNAs and PIWI proteins: From biogenesis to their role in cancer. Gene Rep..

[61] Sellitto A., Geles K., D’Agostino Y., Conte M., Alexandrova E., Rocco D., Nassa G., Giurato G., Tarallo R., Weisz A. (2019). Molecular and Functional Characterization of the Somatic PIWIL1/piRNA Pathway in Colorectal Cancer Cells. Cells.

[62] Jeong H., Park K.H., Lee Y., Jeong A., Choi S., Kim K.W. (2021). The Regulation and Role of piRNAs and PIWI Proteins in Cancer. Processes.

[63] Cheng J., Guo J.M., Xiao B.X., Miao Y., Jiang Z., Zhou H., Li Q.N. (2011). PiRNA, the new non-coding RNA, is aberrantly expressed in human cancer cells. Clin. Chim. Acta.

[64] Cheng J., Deng H., Xiao B., Zhou H., Zhou F., Shen Z., Guo J. (2012). piR-823, a novel non-coding small RNA, demonstrates in vitro and in vivo tumor suppressive activity in human gastric cancer cells. Cancer Lett..

[65] Martinez V.D., Enfield K.S.S., Rowbotham D.A., Lam W.L. (2016). An atlas of gastric PIWI-interacting RNA transcriptomes and their utility for identifying signatures of gastric cancer recurrence. Gastric Cancer.

[66] Yin J., Jiang X.Y., Qi W., Ji C.G., Xie X.L., Zhang D.X., Cui Z.J., Wang C.K., Bai Y., Wang J. (2017). piR-823 contributes to colorectal tumorigenesis by enhancing the transcriptional activity of HSF1. Cancer Sci..

[67] Sabbah N.A., Abdalla W.M., Mawla W.A., Abdalmonem N., Gharib A.F., Abdul-Saboor A., Abdelazem A.S., Raafat N. (2021). PiRNA-823 is a unique potential diagnostic non-invasive biomarker in colorectal cancer patients. Genes.

[68] Weng W., Liu N., Toiyama Y., Kusunoki M., Nagasaka T., Fujiwara T., Wei Q., Qin H., Lin H., Ma Y. (2018). Novel evidence for a PIWI-interacting RNA (piRNA) as an oncogenic mediator of disease progression, and a potential prognostic biomarker in colorectal cancer. Mol. Cancer.

[69] Yin J., Qi W., Ji C.G., Zhang D.X., Xie X.L., Ding Q., Jiang X.Y., Han J., Jiang H.Q. (2019). Small RNA sequencing revealed aberrant piRNA expression profiles in colorectal cancer. Oncol. Rep..

[70] Iyer D.N., Wan T.M.H., Man J.H.W., Sin R.W.Y., Li X., Lo O.S.H., Foo D.C.C., Pang R.W.C., Law W.L., Ng L. (2020). Small RNA Profiling of piRNAs in Colorectal Cancer Identifies Consistent Overexpression of piR-24000 That Correlates Clinically with an Aggressive Disease Phenotype. Cancers.

[71] Mai D., Ding P., Tan L., Zhang J., Pan Z., Bai R., Li C., Li M., Zhou Y., Tan W. (2018). PIWI-interacting RNA-54265 is oncogenic and a potential therapeutic target in colorectal adenocarcinoma. Theranostics.

[72] Müller S., Raulefs S., Bruns P., Afonso-Grunz F., Plötner A., Thermann R., Jäger C., Schlitter A.M., Kong B., Regel I. (2015). Next-generation sequencing reveals novel differentially regulated mRNAs, lncRNAs, miRNAs, sdRNAs and a piRNA in pancreatic cancer. Mol. Cancer.

[73] Xie J., Xing S., Shen B.Y., Chen H.T., Sun B., Wang Z.T., Wang J.W., Lu X.X. (2021). PIWIL1 interacting RNA piR-017061 inhibits pancreatic cancer growth via regulating EFNA5. Hum. Cell.

[74] Law P.T.Y., Qin H., Ching A.K.K., Lai K.P., Co N.N., He M., Lung R.W.M., Chan A.W.H., Chan T.F., Wong N. (2013). Deep sequencing of small RNA transcriptome reveals novel non-coding RNAs in hepatocellular carcinoma. J. Hepatol..

[75] Rizzo F., Rinaldi A., Marchese G., Coviello E., Sellitto A., Cordella A., Giurato G., Nassa G., Ravo M., Tarallo R. (2016). Specific patterns of PIWI-interacting small noncoding RNA expression in dysplastic liver nodules and hepatocellular carcinoma. Oncotarget.

[76] Siegel R., Naishadham D., Jemal A. (2013). Cancer statistics, 2013. CA Cancer J. Clin..

[77] Wild C.P., Weiderpass E., Stewart B.W. (2020). World Cancer Report 2020: Cancer Research for Cancer Prevention.

[78] Zhang X.Y., Zhang P.Y. (2017). Gastric cancer: Somatic genetics as a guide to therapy. J. Med. Genet..

[79] Cui L., Lou Y., Zhang X., Zhou H., Deng H., Song H., Yu X., Xiao B., Wang W., Guo J. (2011). Detection of circulating tumor cells in peripheral blood from patients with gastric cancer using piRNAs as markers. Clin. Biochem..

[80] Bray F., Ferlay J., Soerjomataram I., Siegel R.L., Torre L.A., Jemal A. (2018). Global cancer statistics 2018: GLOBOCAN estimates of incidence and mortality worldwide for 36 cancers in 185 countries. CA Cancer J. Clin..

[81] Herzig D.O., Tsikitis V.L. (2015). Molecular markers for colon diagnosis, prognosis and targeted therapy. J. Surg. Oncol..

[82] Chen H., Xu Z., Liu D. (2019). Small non-coding RNA and colorectal cancer. J. Cell. Mol. Med..

[83] Chu H., Xia L., Qiu X., Gu D., Zhu L., Jin J., Hui G., Hua Q., Du M., Tong N. (2015). Genetic variants in noncoding PIWI-interacting RNA and colorectal cancer risk. Cancer.

[84] Vychytilova-Faltejskova P., Stitkovcova K., Radova L., Sachlova M., Kosarova Z., Slaba K., Kala Z., Svoboda M., Kiss I., Vyzula R. (2018). Circulating PIWI-interacting RNAs piR-5937 and piR-28876 are promising diagnostic biomarkers of colon cancer. Cancer Epidemiol. Biomark. Prev..

[85] Mai D., Zheng Y., Guo H., Ding P., Bai R., Li M., Ye Y., Zhang J., Huang X., Liu D. (2020). Serum piRNA-54265 is a new biomarker for early detection and clinical surveillance of Human Colorectal Cancer. Theranostics.

[86] Tosar J.P., García-Silva M.R., Cayota A. (2021). Circulating SNORD57 rather than piR-54265 is a promising biomarker for colorectal cancer: Common pitfalls in the study of somatic piRNAs in cancer. RNA.

[87] Qu A., Wang W., Yang Y., Zhang X., Dong Y., Zheng G., Wu Q., Zou M., Du L., Wang Y. (2019). A serum piRNA signature as promising non-invasive diagnostic and prognostic biomarkers for colorectal cancer. Cancer Manag. Res..

[88] Gu X., Wang C., Deng H., Qing C., Liu R., Liu S., Xue X. (2020). Exosomal piRNA profiling revealed unique circulating piRNA signatures of cholangiocarcinoma and gallbladder carcinoma. Acta Biochim. Biophys. Sin..

[89] Adamska A., Domenichini A., Falasca M. (2017). Pancreatic Ductal Adenocarcinoma: Current and Evolving Therapies. Int. J. Mol. Sci..

[90] Rawla P., Sunkara T., Gaduputi V. (2019). Epidemiology of Pancreatic Cancer: Global Trends, Etiology and Risk Factors. World J. Oncol..

[91] El-Serag H.B. (2012). Epidemiology of Viral Hepatitis and Hepatocellular Carcinoma. Gastroenterology.

[92] Llovet J.M., Kelley R.K., Villanueva A., Singal A.G., Pikarsky E., Roayaie S., Lencioni R., Koike K., Zucman-Rossi J., Finn R.S. (2021). Hepatocellular carcinoma. Nat. Rev. Dis. Prim..

[93] Tang X., Xie X., Wang X., Wang Y., Jiang X., Jiang H. (2018). The combination of piR-823 and eukaryotic initiation factor 3 B (EIF3B) activates hepatic stellate cells via upregulating TGF-β1 in liver fibrogenesis. Med. Sci. Monit..

[94] Rakić M., Patrlj L., Kopljar M., Kliček R., Kolovrat M., Loncar B., Busic Z. (2014). Gallbladder cancer. Hepatobiliary Surg. Nutr..

[95] Hickman L., Contreras C. (2019). Gallbladder Cancer: Diagnosis, Surgical Management, and Adjuvant Therapies. Surg. Clin. North. Am..

[96] Espinoza J.A., Bizama C., García P., Ferreccio C., Javle M., Miquel J.F., Koshiol J., Roa J.C. (2016). The inflammatory inception of gallbladder cancer. Biochim. Biophys. Acta.

[97] Bertran E., Heise K., Andia M.E., Ferreccio C. (2010). Gallbladder cancer: Incidence and survival in a high-risk area of Chile. Int. J. Cancer.

[98] Hundal R., Shaffer E.A. (2014). Gallbladder cancer: Epidemiology and outcome. Clin. Epidemiol..

[99] Buchegger K., Silva R., López J., Ili C., Araya J.C., Leal P., Brebi P., Riquelme I., Roa J.C. (2017). The ERK/MAPK pathway is overexpressed and activated in gallbladder cancer. Pathol. Res. Pract..

[100] Keller S., Ridinger J., Rupp A.K., Janssen J.W., Altevogt P. (2011). Body fluid derived exosomes as a novel template for clinical diagnostics. J. Transl. Med..

[101] Cheng L., Sharples R.A., Scicluna B.J., Hill A.F. (2014). Exosomes provide a protective and enriched source of miRNA for biomarker profiling compared to intracellular and cell-free blood. J. Extracell. Vesicles.

[102] Quek C., Bellingham S.A., Jung C.-H., Scicluna B.J., Shambrook M.C., Sharples R.A., Cheng L., Hill A.F. (2017). Defining the purity of exosomes required for diagnostic profiling of small RNA suitable for biomarker discovery. RNA Biol..

[103] Dowdy S.F. (2017). Overcoming cellular barriers for RNA therapeutics. Nat. Biotechnol..

[104] Winkle M., El-Daly S.M., Fabbri M., Calin G.A. (2021). Noncoding RNA therapeutics—Challenges and potential solutions. Nat. Rev. Drug Discov..

[105] Ning B., Yu D., Yu A.-M. (2019). Advances and challenges in studying noncoding RNA regulation of drug metabolism and development of RNA therapeutics. Biochem. Pharmacol..

